# Consent2Share: an integrated broad consenting process for re-contacting potential study subjects

**Published:** 2016-10-10

**Authors:** R Peter Iafrate, Gloria P Lipori, Christopher A Harle, David R Nelson, Timothy J Barnash, Patricia T Leebove, Kathleen A Adams, Debbi Montgomery

**Affiliations:** 1 *Institutional Review Board, University of Florida, Gainesville, Florida, United States*; 2 *Operational Planning & Analysis, University of Florida Health and University of Florida Health Sciences Center, Gainesville, Florida, United States*; 3 *Department of Health Policy and Management, Indiana University, Indianapolis, Indiana, United States*; 4 *Department of Medicine, University of Florida, Gainesville, Florida, United States*; 5 *Practice Management Applications, UF Health Physicians, University of Florida, Gainesville, Florida, United States*; 6 *Medical Specialties and Transplant Clinic, UF Health Physicians, University of Florida, Gainesville, Florida, United States*; 7 *Information Technology, UF Health, Gainesville, Florida, United States*

**Keywords:** consent, recruitment, research subjects, clinical research studies, contact registries, global consent, electronic consent

## Abstract

**Background and Aim:** Obtaining sufficient subjects into research studies is an ongoing barrier to conducting clinical research. Privacy rules add to the complexity of identifying qualified study subjects. The process described facilitates consent of patients coming to their clinically scheduled appointments who are asked to consent to having researchers review their electronic medical records (EHR), and if they meet study criteria for future research, being contacted by those researchers and asked if they wish to be involved in a research project.

**Methods:** An interdisciplinary group representing the Institutional Review Board (IRB), Information Technology (IT), Hospital, University and Research developed an initial paper then electronic method to consent all patients attending a medical subspecialty clinic. All consent data are integrated to the EHR to facilitate linking to clinical information.

**Results:** Although the paper consenting method resulted in over an 80% “yes” rate of consent, it was complicated by significant procedural challenges which prevented scalability. Revising the process has resulted in nearly 28,000 patients consenting in a 3 year period and in 20 IRB approved protocols using subjects who agreed to Consent2Share.

**Conclusions:** A multi-disciplinary effort is essential to develop a successful electronic based, integrated process to assist investigators and patients to facilitate study subject accrual.

**Relevance for patients:** Consent2Share more efficiently assists researchers in identifying and contacting potential study subjects that meet entrance criteria. The process provides a model to comply with the proposed Notice of Public Rule Making (NPRM) where institutions will be strongly encouraged to develop broad research consent procedures.

## 1. Introduction

A limited ability to recruit sufficient subjects into research studies is an ongoing barrier to the efficient production of generalizable clinical research and has been estimated to cost up to one million dollars per day [1,2]. Patients often want to be involved in clinical research but do not know what studies are available or how they can be accessed [3]. Balancing the needs of the research community for access to patient medical data and protecting the integrity of patients’ privacy has presented an ongoing challenge to establishing core principles of oversight at research focused medical institutions [4-7].

Defining the parameters of patient consent is a key priority at academic research institutions. Patient consent for research can fall within two archetypes: “broad consent” vs. “categorical consent.” Both describe a process of asking patients to agree prospectively to have their health records available to researchers within appropriate oversight. Whereas broad consent stipulates agreement for being contacted for any future research; categorical consent is tied to specific studies [5,8]. There are substantial advantages of favoring broad consent. Broad consenting practice engenders greater efficiencies and cost effective scalability of recruitment, access to real time medical records, a greater probability of documenting rare disease, and the increased likelihood of capturing a more diverse population [8-9]. The process of broad consent is complex and dynamic. The eMERGE (electronic Medical Records and Genomics) Network of DNA repositories has served as an excellent laboratory to explore the sensitive features of consenting practice [4,10-11]. To underscore the dynamic nature of consent in general, investigators question when and how participants can “opt out.” [4,8,11-12] Determining how and when the consenting form is provided and the ability of participants to fully comprehend the meaning of consent adds to the complexity of the process [7,12-15]. Furthermore, patients have pre-conceived knowledge and concerns about the meaning of consent and have preferences for the process of consenting [5,14-16]. Finally, how is the role of the investigator defined to the participant when they were recruited while seeking routine medical care [4,8,11-12]. Clearly, the role of oversight and governance must serve as the primary concern for the research community [6].

This paper outlines a process and practice for obtaining broad consent from patients who seek treatment from the University of Florida Health clinical practice. This results in a contact registry for researchers to use to identify potential study subjects that have given consent and authorization to be approached for future research efforts. In addition to addressing the regulatory and ethical concerns associated with participant recruitment and data management, the immediate challenge facing researchers is threefold: 1) the effort devoted to recruiting patients for potential studies often does not yield sufficient numbers or diversity; [8] 2) developing a protocol for each individual study and approaching patients with multiple consent forms to ensure that patients fully understand the scope of their consent for each is time consuming, repetitive, and likely to be of inconsistent quality from study to study; [15,17-18] and 3) researchers do not have the infrastructure and the support to appropriately maintain a database integrated with patients’ electronic health record (EHR) to ensure that up-to-date information is maintained on potential study subjects, and is integrated with other databases and searchable based on study criteria.

With the recent release of a Notice of Public Rule Making (NPRM) [19] on potential major changes to the Health and Human Service Common Rule, institutions will be strongly encouraged to develop broad research consent procedures that include tracking patients’ choices for opting in or not. The proposed changes to the Common Rule encourage increased use of “broad consent” of patients for research use of their biospecimens and identifiable data. Within this broad consent framework, the basic required elements of informed consent, found in 45 CFR 46.116(a), would remain the same. These include, but are not limited to, describing purpose, procedures, risks, benefits, alternatives, and confidentiality issues. Additionally, the regulations require that each subject’s involvement be voluntary. However, broad consent, which most likely will occur during routine clinical encounters, is envisioned as a useful mechanism for making research easier to conduct while still preserving the voluntary nature of research participation. In particular, broad consent processes may help alleviate researcher challenges in recruitment, study protocol management, and data management infrastructure. However, in a review by Garrison et al, they found that many people do not favor broad consent for either research itself or for research and subsequent wide data sharing, particularly when other types of consent (ie: study-by-study or tiered consent in which participants are given a set of options allowing them to select how they want to participate in the research.) were available [5]. Unlike most of the 48 studies reviewed by Garrison et al., subjects agreeing to the Consent2Share process only agree to be contacted to hear about a future research studies, and not to participate in any future study without study specific consent.

In recent years, the University of Florida Health Science Center and the University of Florida Institutional Review Board (IRB) have chosen to prioritize the challenges outlined above. In this article, we describe the initial rollout, lessons learned, and evolution of the University of Florida Health System’s “Consent2Share” recruitment program, which was designed to help overcome these challenges. Consent2Share advances on more traditional procedures of maintaining a registry, whether a simple database that includes only contact information, or a more sophisticated regional or national registry that includes health information that can be searched to find individuals who meet specific study criteria [20]. Often, the restrictions with these registries result from patients’ consent for specific studies or from a time-limited consent. Therefore, researchers have access to a much smaller set of potential study participants. Instead, the Consent2Share program asks patients’ permission to be re-contacted for potential participation in future research studies and does not limit the patients’ participation to studies that focus on the condition for which the patient was seeking treatment at the time of recruitment. Developing a single recruiting effort that has no limitation on what study patients may be recruited for and no particular time frame substantially increases the number and diversity of patients to which the IRB would allow access.

Routine clinical encounters provide a valuable touch point where a trusted entity can approach a large number of patients regarding potential participation in clinical research. The study leveraged opportunities to recruit while patients sought treatment in a UF clinical practice. However, often busy clinical workflows and clinician and staff time constraints also pose barriers to approaching and consenting patients to participate in research studies [21]. The following is a summary of the iterative process undertaken by the UF Health system to recruit patients, integrate the IRB approval process, and diminish the burden on researchers of recruiting patients. Strict oversight by the IRB ensures the safeguarding of the principles the IRB implements to protect human subjects. This article outlines the governance structure necessary for this comprehensive program, the technological requirement, the initial pilot, the self-audit evaluation, and the necessary process re-design.

## 2. Stakeholders and program governance

Ethical practice of broad consent is a primary concern for the medical research community, especially in providing consistent protocol, assessing participant attitude, and preserving patient privacy and ability for opting out [4-6,22-23]. To ensure ethical best practice, the program is governed by stakeholders connected across the university and the health system. These stakeholders represent UF Health leadership, the IRB, the departments that provide analytic and informatics support, the departments that manage information technology, and the departments responsible for the collection of patient records, data storage, and information technology architecture. This multidisciplinary group consists of the UF Health Office of the Chief Data Officer (OCDO), the Clinical and Translational Sciences Institute, the information technology team at the UF Health Sciences Center and the Health Sciences Center faculty, and the IRB chairperson. Each stakeholder group brought their expertise and unique outlook to the design of the program to ensure that the outcome represents the best practices for how data are collected, stored, used, and protected. This group governs the use of clinical data for research, and therefore represents the natural domain to conceptualize, plan, and oversee the capture and use of Consent2Share information, all under the federal regulations governing respect for human subjects. The team met weekly during the implementation and expansion phases of the program. With their expertise, they guide the development and implementation of data tools to access institutional data for research, clinical, and educational activities in a secure and Health Insurance Portability and Accountability Act (HIPAA)-compliant environment.

## 3. Institutional Review Board approval of the integrated data repository

**Figure 1:**
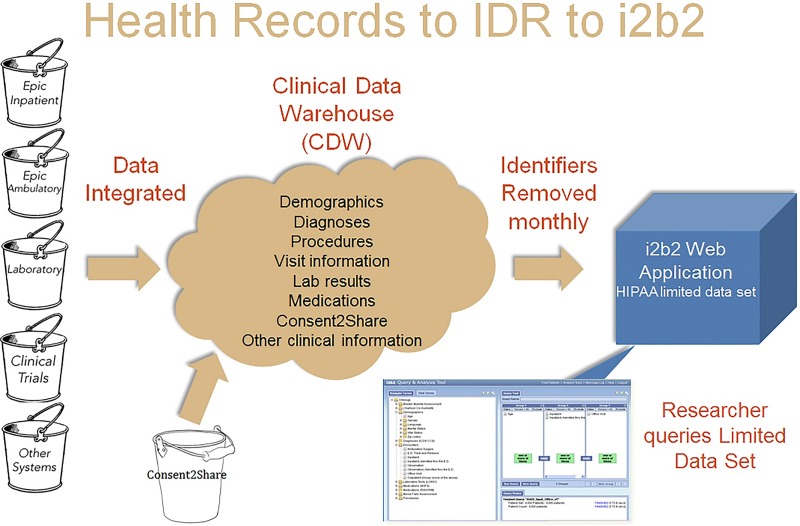
Consent process added to i2b2 for cohort discovery.

To help serve the needs of the Health Science Center at the University of Florida, the integrated data repository (IDR) was created to serve as a common source of information to be used by clinicians, executives, researchers, and educators. The IDR consists of a Clinical Data Warehouse (CDW) that aggregates data from the various clinical and administrative information systems, including the Epic EHR (Figure 1). The CDW contains demographics, nearly 15 million inpatient and outpatient clinical encounter data, diagnoses, procedures, lab results, medications, select nursing assessments, co-morbidity measures, and select perioperative anesthesia data.

Because the IDR’s CDW cannot be directly accessed for research purposes, in 2011, a “UF Health IDR” protocol was submitted to the UF IRB as a research tissue and data bank. Each month, a query of the CDW is run to extract a HIPAA-compliant “Limited Data Set” that replicates data from the CDW. This Limited Data Set, which by definition is de-identified except for procedure dates and zip code information, can be queried by researcher from their private desk top computer, via i2b2. Informatics for Integrating Biology and the Bedside (i2b2) provides a scalable informatics framework with a search and query function that allows researchers access to the IRB-approved limited dataset through an Internet browser [24]. Because the IDR has established a safe, secure, consistent, and reliable method for protecting patient data and has an IRB-approved search function for cohort discovery, it was the ideal environment to store and provide access to the potential recruitment data of patients agreeing to the Consent2Share process.

**Figure 2:**
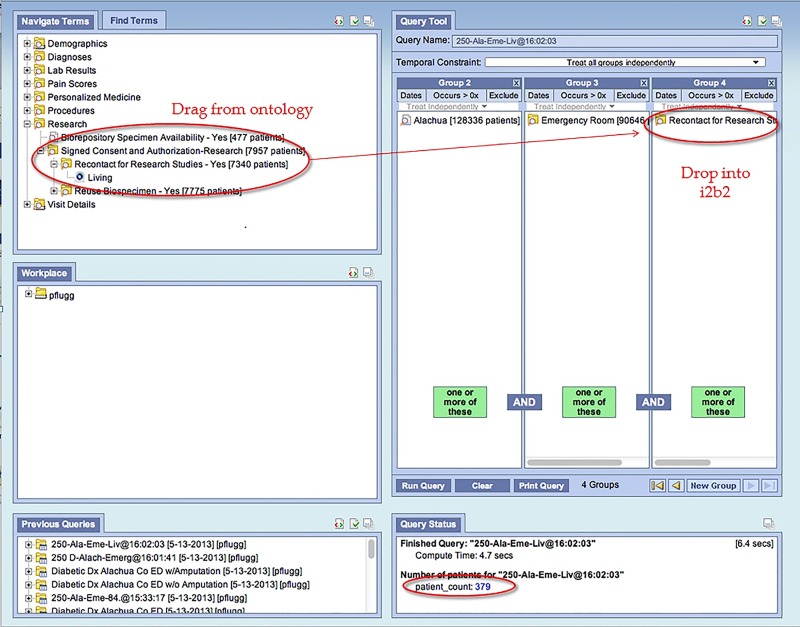
Screen shot of i2b2 query and cohort result.

Using i2b2 when developing potential study protocols, researchers can conduct queries based on their inclusion exclusion criteria prior to IRB approval. These criteria are dragged from a provided ontology of diseases, labs, medications, etc., and dropped into the i2b2 query format (Figure 2). Within seconds, the researcher will know how many patients within the UF Health Science Center meet the proposed inclusion\exclusion criteria. Only the number of potential subjects is provided, along with de-identified demographic information. If the researcher considers the number of potential subjects is too small to conduct their research, criteria can be adjusted and queries re-run until a reasonable potential study subject pool exists. Such a query will identify cohort counts as researchers prepare grant proposals, plan clinical trials, write IRB protocols, and conduct other preparatory research activities. One of the criteria available can include whether patients have agreed to be re-contacted via the Consent2Share process. Thus, if the study being designed will require the researcher to contact the potential subject, that criteria is also dropped into the i2b2 query. The researcher is not given the contact information at this point, only the number of potential subjects that meet inclusion\exclusion criteria and have agreed to be contacted for future research projects.

Once the researcher determines that there are enough potential study subjects in the cohort that meet study criteria, he/she applies for IRB protocol approval. Once the protocol is IRB approved, the researcher then provides the protocol-specific query to an honest broker (a neutral intermediary between researchers and the identifiable data) \data analyst, who runs the query again against the identifiable data base, and then provides a file of the potential subjects to the researcher across a secure platform, including contact information for those who have agreed to be re-contacted.

## 4. Pilot

### 4.1. Initial consent capture process (Consent2Share)

To augment the “UF Health IDR” protocol with patient consent information, an IRB revision was submitted and approved to allow for the consenting of patients for re-contacting as part of their routine clinic visits. The original goal of this effort was to give patients who wanted to be considered for future research protocols a way to provide their name to researchers to let them know they are interested, and to potentially improve recruitment into research studies. The consent form needed to be as short as possible without neglecting any human subject or privacy regulation. In the original three-page consent form, subjects were consenting to the following:

1. “Store some of your excess blood or excess tissue that is not needed for your medical treatment and store your identifiable medical information about your blood or excess tissue in a secure location. These tissue samples could be used to help answer research questions such as treating, diagnosing, or preventing certain diseases.”
2. “To contact you by phone, mail, or email about being part of a new research study at UF Health. If you are contacted, you will be told about a specific research study; you can choose whether or not to involve yourself.”

It was important that the initial test site take place in a clinic with a strong clinical champion and where the patients were all adults, and where most patients were competent to consent for themselves. For simplicity and the protection of vulnerable patients, in the pilot stage of the program, the selection criteria excluded children or incompetent adults. We decided that we will eventually expand to include children since the software could identify when the child turned 18, and thus the now adult patient would need to be reconsented. We decided at this point we will never include incompetent adults, since identifying if those individuals regain competency and thus would require a reconsent, would be too difficult to accomplish with the number of patients agreeing to Consent2Share. The final test site chosen was the Internal Medicine and Medical Specialties clinic which includes Hepatology, Infectious Disease, Gastroenterology, Endocrinology, Nephrology, Pulmonary, Rheumatology, Internal Medicine (primary care), Lung Transplant, Renal Transplant, Liver Transplant, and Transplant Surgery. Moreover, a strong physician champion practices in this clinic, Dr. David Nelson, PI, of the CTSAgrant.

The IT team configured the Epic EHR system to electronically record the decisions a patient made regarding the Consent2 Share effort. The IRB-approved consent form was loaded into Epic, allowing a patient-specific research consent form to be printed before that patient’s clinic visit. The form contained a bar code of patient-specific information at the top of each page.

The IRB Chairperson and co-PI for the IDR protocol provided a training course to all admission clerks working at the Internal Medicine and Medical Specialties clinic. Once clinic registration was complete, the role of the admission clerks was to provide the patient with a Consent2Share flier and a copy of the three-page, patient-specific consent form, which patients were then given time to review. After a patient returned his/her consent form, the admission clerk would record the decision of the patient directly into the EHR as discrete data and store the signed consent form for future scanning into OnBase, the document imaging system integrated within Epic. Subsequently, the discrete consent information (i.e., yes or no only) was extracted from the EHR and loaded into the IDR so that researchers could query the data via i2b2 along with other inclusion\exclusion criteria.

The process in Epic was designed to do the following, based on the patient’s choice:

1. If the patient chose “Yes” or “No” to participate in either or both of the options, then no Consent2Share form would print on subsequent clinic visits.
2. If the patient verbally told the admission clerk “No,” this was recorded by the admission clerk into Epic, and no Consent2Share form would print on subsequent clinic visits.
3. If the patient failed to return the Consent2Share form, then the Consent2Share form would print at the next clinic visit.

For additional patient protection and regulatory compliance, a copy of the Consent2Share consent form was offered to any patient who agreed to participate in the Consent2Share effort. Furthermore, if the patient had questions about the Consent2Share process, they could simply not return the consent form and ask questions of the physician with whom they had an appointment. The consent form also includes a hotline number that patients can call 24\7 with questions. Hotline attendants are provided a Frequently Asked Questions (approved by the IRB) regarding the process. Should the patient have additional questions, he/she is referred to a Patient Advocate within the university’s Clinical and Translational Science Institute. Patients can also call the hotline if they changed their mind regarding their participation in Consent2Share. A process is in place in which the Patient Advocate calls that patient, and if he/she wishes to stop participation, the Patient Advocate electronically changes the answer from “Yes” to “No” and documents the conversation. Out of the nearly 30,000 subjects who agreed to Consent2Share, this has occurred only six times in the past four years.

### 4.2. Consent re-use for research process

UF researchers can use the i2b2 system to query the “UF Health IDR” for the number and basic demographics of patients who meet their inclusion and exclusion criteria. Once the Consent2Share program was implemented, researchers could limit their queries to determine how many patients who had agreed to be re-contacted about research studies (Figure 2) also met inclusion criteria for that particular study. After using i2b2 and determining there are enough potential study subjects that meet their inclusion\exclusion criteria, researchers can apply for IRB approval to obtain more extensive data from the CDW With IRB approval, the OCDO team who are certified by the University as “Honest Brokers” will provide a research data set that can include contact information for participant recruitment activities.

As part of the IRB approval, when investigators contact potential study subjects from the Consent2Share list either by mail, email, or phone, the following introduction must be used:

“Dear <potential subject name>,

My name is <name> and I <title or study staff relationship> from <UF or Shands>. I am contacting you to see if you are interested in participating in a research study <describe the topic briefly [e.g. On diabetes]>. During a past clinic visit, you signed a consent form telling us you were interested in being contacted for future research that you might qualify for.

*The following is information about a research study on <state topic [e.g. Diabetes]> that you might qualify for. If you are interested, please read the following and <include the process for the subject to complete something, call you back, etc.*>“

Early on in this process, some patients who were contacted by researchers to determine if they were interested in a particular study did not recall that they had signed a consent to be re-contacted regarding unknown future research. Therefore, the follow up script above includes language that helps to inform the potential subject how the caller came to have his/her contact information, and to remind them of their agreement to the Consent2Share process. The use of the contact information for any potential subject is limited to each request approved by the IRB. Since the limited data set that researchers have access to through i2b2 is re-run each month, researchers have no way to collect further information regarding subjects via i2b2 since there is no way to identify a particular subject.

## 5. Results

### 5.1. Self-audit

After five months of the Consent2Share program pilot, the IDR team conducted a self-audit of the process and the resultant consent forms. At that point, nearly 8,000 patients had consented, 78% “Yes” for the re-contact and 83% “Yes” for tissue collection, which is similar to what others have found using various types of broad consent (Ref. Garrison et al). In a 10% statistically designed random audit, there were several unexpected results, including 75 missing consent forms, forms where subjects signed on the wrong line, forms where subjects circled both “Yes” and “No,” and forms where the subject did not initial next to “Yes” or “No.”

An assessment was done of the processes and many of the missing consent forms were found to be electronically linked to the wrong patients, scanned such that pages did not appear contiguously, etc. During the audit month, the number of consented subjects reached approximately 10,000. Due to the errors, it was clear that the process was flawed and was therefore voluntarily suspended the Consent2Share part of the “UF Health IDR” protocol and reported these issues to the IRB.

**Figure 3:**
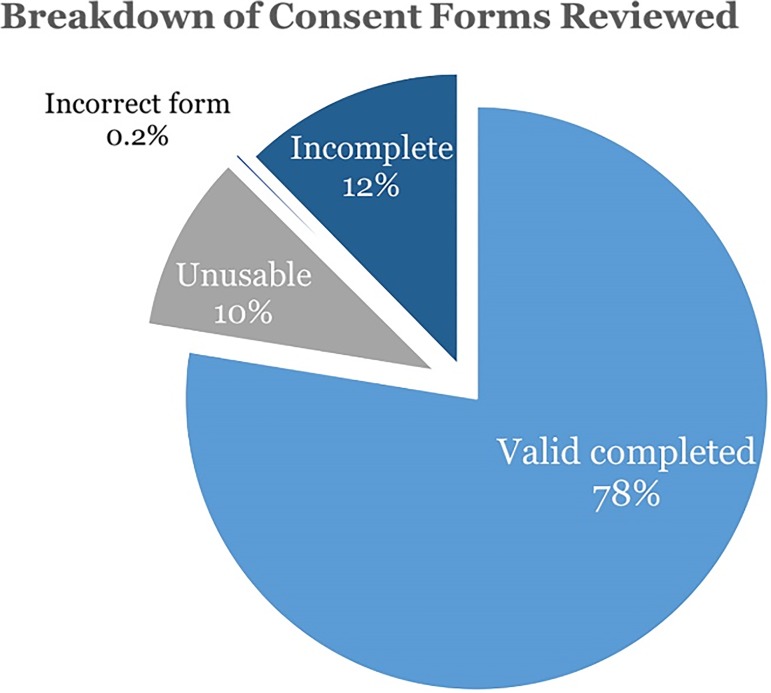
Breakdown of consent forms reviewed.

Over the subsequent four months, new staff joined the team and conducted a 100% review of all 10,000 consent forms. Only those deemed to be a proper consent with accurate information within Epic were kept; all others were re-set in Epic to remove any information. The results from the review of the nearly 10,000 consents (Figure 3):

- 10,460 consent forms reviewed
- The IRB Principle Investigator and IRB determined which consent forms to be determined as “valid” based on criteria
- 77.6% were complete valid consent forms (8,118)
- 9.7% were complete invalid or unusable consent forms (1,032)
- 0.2% were the wrong consent forms and were removed (25)
- 12.3% were incomplete consent forms – scanned pages not connected to the correct EHR (1,291)
  - 462 complete and valid consent forms were assembled
  - 47 complete and invalid consent forms were assembled

### 5.2. Process re-design

The team adopted an iterative developmental approach, making adjustments and fine tuning the process, to minimize errors in the future. The following steps were included in the corrective action plan submitted to and approved by the IRB:

- Eliminated the request for leftover tissue collection to simplify the process and the consent form.
- All admission clerks were re-trained on how to interact with potential subjects and how to record data in Epic.
- The consent form was simplified and reformatted to minimize the possibility of subject errors.
- Potential subjects were given a new brochure that explained the reason for asking them to enroll in the Consent2Share program, and they received two copies of the consent form up front: one for them to keep if they agreed to participate and one to complete and return.
- The clinic staff scanned the consent forms and were able to determine if there was an error prior to submitting it. Previously, the forms were sent to a central data office and scanned along with clinical records.
- All newly enrolled subject consent forms and recorded data were verified and confirmed the following day by trained staff in the Office of Data and Analytics. Each application was reviewed for the following:
  - That the consent form was “valid.”
  - That the consent form was attached to the correct patient.
  - That the choices made were recorded correctly.
  - The individual conducting the questions and answers marked each verified record that the information could be sent to the IDR.

The changes resulted in a much smoother and simpler process for the patient, the admissions staff, and the staff from the office of the Chief Data Officer. Continuous checking of each form confirmed that the revised process resulted in the accurate recording of information and usable potential study subjects. Over the next year, the process continued smoothly. To date, nearly 28,000 study subjects have completed a consent form, with a positive consent to be re-contacted at a rate of 83%. However, to further protect the integrity of the process, before any patient contact information is released to a researcher through the program, an additional 100% manual audit is conducted on the consent forms for the requested patients.

### 5.3. eConsent2Share

The UF Health Consent2Share process has been in place for almost four years. In that time, there have been many requests to expand this pilot to other clinics. However, the IDR steering committee decided there would be no expansion until an electronic consent process, integrated with Epic, was available. With the insight gained from the initial paper Consent2Share process, the team strongly believed that an electronic consenting process (eConsent) would further minimize errors and substantially decrease the effort for both the front-end clinical staff and the auditing staff.

**Figure 4:**
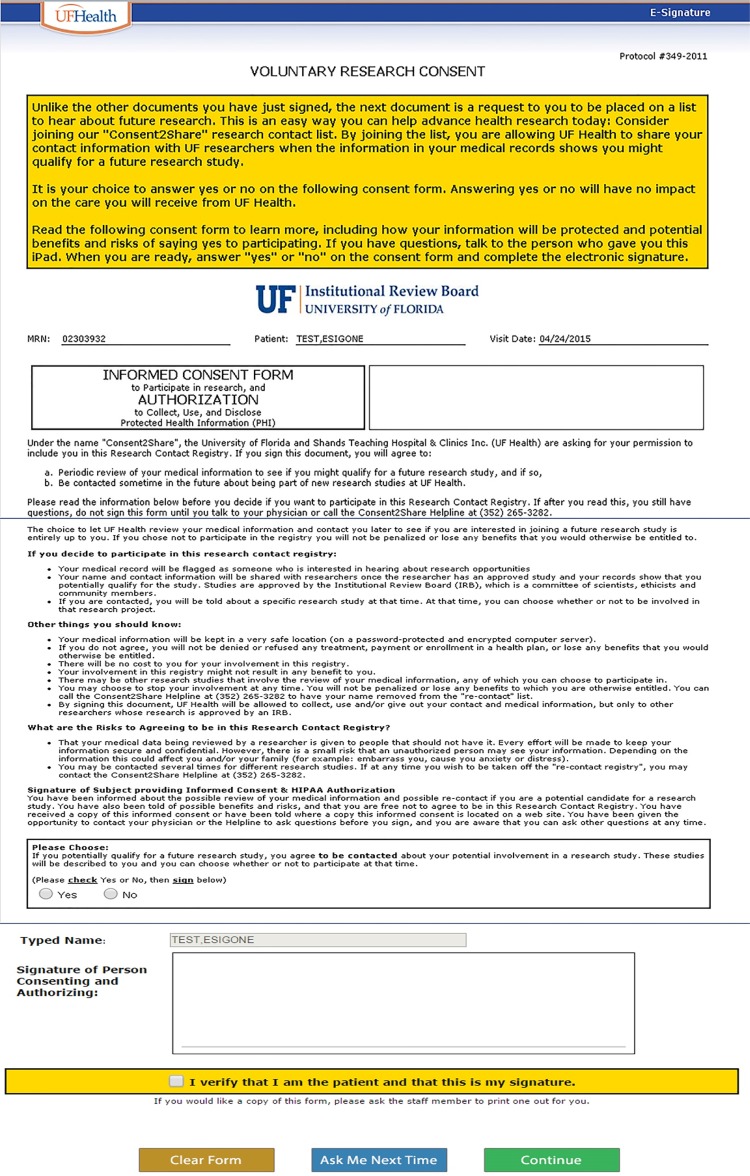
Consent2Share eSignature screens.

In May of 2015, an eConsent form and process was launched after approval by the IRB. In synch with all clinical consent forms being electronic, the eConsent2Share was incorporated into an iPad format. Epic was configured to always place the Consent2Share consent document as the last consent form a patient would see. It is distinguished by a bright yellow box indicating that the following consent was for research and was optional (Figure 4).

The eConsent2Share process eliminated the problems of subjects completing the consent document incorrectly or not clearly choosing “yes” or “no.” Electronic copies of consent forms are immediately attached to the correct patient and electronically filed in the host EHR eliminating scanning errors. We no longer have to validate each consent form, although each time an investigator is approved to contact patients that agreed to Consent2Share, a data analyst reviews only those eConsents to insure they were completed correctly and mainly to determine if the patient is deceased. Although the eConsent2Share eliminated most of the process issues with this program, including decreaseing the effort by the admission staff, it did not have any effect on the rate of those subjects consenting to this effort. The program has been expanded to two new practice clinics; a primary care clinic, and a cardiovascular clinic.

## 6. Discussion

Within the research community at UF, the awareness of the data in the IDR and the use of i2b2 is increasing. Since adding information on patients that have agreed to enroll in Consent2Share to i2b2, completed in January 2013, 41 investigators had defined their search query to include patients who have agreed to be re-contacted. Between January and August 2015, 23 investigators have used Consent2Share criteria in their i2b2 queries. Of these 41 self-service queries, the OCDO has received 20 IRB-approved protocols for identifying patients for potential studies. Because researchers can query the database to determine whether there are sufficient numbers of patients meeting specific clinical criteria before submitting their application to the IRB, they can save substantial time in the life cycle of a medical research study. Additionally, investigators can search for the number of potential patients to qualify as part of a grant application. Often, funding agencies want assurance that the study will be able to recruit adequate numbers. The i2b2 software can provide that type of data quickly and at no expense to the researcher.

Garrison et al. extensive review of literature reported on various factors that may impact rates of patient recruitment; specifically, demographics and attitudinal disposition. [5] The Consent2Share process outlined in this paper with its attention to patients in their treatment location and personal attention by staff yields a relatively high rate of consent. Although the average “yes” rate for those approached to Consent2Share has been above 80% in our initial clinic, the “yes” rate in our second clinic has settled into about a 50% rate. We have not studied the reason for this, we postulate it results from the intial clinic seeing older, sicker subjects who may be more motivated to be involed in research, while the newer location is a primary care clinic in a relatively affluent subdivision. However, a follow-up study to evaluate non-consent and report on the specific attributes of the consenting population would contribute to our knowledge in this area, and the continuous quality improvement of consenting practice.

In the coming years as we expand to all clinics and the inpatient hospital, we will no doubt experience additional challenges involving pediatric patients, psychiatry patients, pregnant patients, etc. Our consent form will be revised allowing for pop-up windows where potential subjects can click on to obtain additional information related to research and this consent if they choose. Tissue samples will be targeted toward certain patients based on approved studies, using data already available within the EPIC EHR. In addition, a revision to the iDR\Consent2Share protocol will be submitted to allow follow-up with researchers to determine the success rate of enrolling a study subject in a future study when using the Consnet2Share method of obtaining potential study subjects’ contact information.

Those using i2b2 can link information through a web-based software network called SHRINE (Shared Health Research Informatics Network) to allow researcher from one institution query information from multiple other institutions. Although the University of Florida is involved with a SHRINE effort, we hope in the future other institutions develop a similar Consent2Share effort which could then allow cross institutional contacting of potential study subjects.

The process of recruiting patients within the clinical setting for future re-contact, those who are not limited by specific medical conditions, has the substantial advantage of creating access to a larger and more diverse set of patients who may qualify for a greater variety of research studies. In the initial self-audit evaluation, the process faced several challenges to which the Consent2Share team responded and made the modifications necessary to ensure greater reliability with simultaneous attention to patient choice and autonomy. To further reduce errors when enrolling potential study participants, the team introduced an electronic process that eliminated the possibility of the most common problems associated with recruitment. This iterative process has led to an innovative approach to recruiting patients for research while safeguarding the human subjects that will ultimately benefit from medical research.

## Technological foundation

Technologically, the Consent2Share program relies on the institution’s underlying clinical and research data infrastructure. The UF Health Office of the Chief Data Officer (OCDO) manages the UF Health Integrated Data Repository (IDR). The IDR was created to serve as a common source of information to be used by clinicians, executives, researchers, and educators. The IDR consists of a Clinical Data Warehouse (CDW) that aggregates data from the various clinical and administrative information systems, including the Epic EHR (Figure 1). The data warehouse contains demographics, inpatient and outpatient clinical encounter data, diagnoses, procedures, lab results, medications, select nursing assessments, co-morbidity measures, and select perioperative anesthesia data.

The IDR oversees a cohort discovery tool that gives investigators a window to the clinical data that are available in the data warehouse. Informatics for Integrating Biology and the Bedside (i2b2) provides a scalable informatics framework with a search and query function to the clinical data that allows users access to an IRB-approved limited dataset through an Internet browser [24]. Because the IDR has established a safe, secure, consistent, and reliable method for protecting patient data and has an IRB-approved search function for cohort discovery, it was the ideal environment to store and provide access to the potential recruitment data of patients agreeing to the Consent2Share process. Addressing the challenges of data transfer and the possible failure in maintaining data integrity is fundamental to ensuring that broad consent practice is not challenged. To minimize risk, honest brokers trained in both data security, informatics, and data programming are integral to the process [25-26]. The IDR follows honest broker best practice and strict guidelines for transferring data to research investigators.

The IDR, by curating and harmonizing patient EHRs, provides the technological infrastructure to ensure institution-wide access to information on patient willingness to be contacted regarding research. After the Consent2Share consent is obtained in a process that aligns with regular clinical workflows, consent information is extracted and loaded into the IDR. Researchers can then use the i2b2 tool to execute cohort discovery queries to identify only the number of patients who meet specific inclusion and exclusion criteria and have agreed to be re-contacted.
